# Association between triglyceride-glucose index and risk of endometriosis in US population: results from the national health and nutrition examination survey (1999–2006)

**DOI:** 10.3389/fendo.2024.1371393

**Published:** 2024-05-28

**Authors:** Penglin Liu, Yixiao Wang, Xuechao Ji, Wenzhi Kong, Zangyu Pan, Chunyu Xu, Yuning Geng, Jinwei Miao

**Affiliations:** Department of Gynecologic Oncology, Beijing Obstetrics and Gynecology Hospital, Capital Medical University, Beijing Maternal and Child Health Care Hospital, Beijing, China

**Keywords:** endometriosis, triglyceride-glucose index, insulin resistance, cross-sectional study, National Health and Nutrition Examination Survey

## Abstract

**Background and Aims:**

The association of the triglyceride-glucose (TyG) index, a promising novel biomarker for insulin resistance, with the risk of endometriosis has not been investigated to date. This nationwide study aimed to explore the association between the TyG index and the endometriosis risk.

**Methods:**

Data were obtained from the National Health and Nutrition Examination Survey (1999–2006). Female participants who provided complete data on the TyG index and endometriosis were enrolled in the analysis. Multivariate logistic regression analyses were utilized to assess the association of the TyG index with endometriosis, adjusted by multiple potential confounders. Meanwhile, in-depth subgroup analyses were conducted.

**Results:**

A total of 1,590 eligible participants were included, among whom 135 (8.5%) women were diagnosed with endometriosis. The fully adjusted multivariate logistic model showed TyG index was significantly associated with the endometriosis risk (odds ratio [OR]_Q4 versus Q1_ 2.04, 95% confidence interval [CI]: 1.15–3.62; *P* for trend=0.013). In subgroup analyses, the significantly positive association between TyG index and the risk of endometriosis was also found in parous women (OR_Q4 versus Q1_ 2.18, 95% CI: 1.20–3.96), women without diabetes (OR _Q4 versus Q1_ 2.12, 95% CI: 1.19–3.79), women who smoke currently (OR _Q4 versus Q1_ 3.93, 95% CI: 1.33–11.58), women who drink currently (OR _Q4 versus Q1_ 2.54, 95% CI: 1.27–5.07), and in women who use oral contraceptives (OR _Q4 versus Q1_ 1.91, 95% CI: 1.04–3.51). Additionally, significantly increasing trends in the odds of endometriosis across the quartiles of the TyG index were observed in the above-mentioned subgroups (all *P* for trend<0.05).

**Conclusions:**

This population-based study found that a higher TyG index, representing an increased level of insulin resistance, was associated with a higher risk of endometriosis among the US population. Our findings suggested TyG index might be a promising tool for the risk assessment of endometriosis. Prospective studies are warranted to further verify these findings.

## 1 Introduction

Endometriosis, as a chronic, systemic gynecologic disease, is characterized by the implantation of endometrial-like tissue outside the uterine cavity, commonly affecting the pelvic cavity and ovaries (1). It is estimated that endometriosis affects approximately 5–10% of women in their reproductive years and exerts significant psychological and physical effects on the quality of life (1–3). However, current treatments for endometriosis including surgical removal of lesions and drug therapy both have limited efficacy (4). The high occurrence and recurrence rates lead to tremendous healthcare expenses for long-term management, highlighting the urgent need to develop robust novel biomarkers for the risk assessment, diagnosis and monitoring of disease progression.

The exact pathological mechanism of endometriosis remains not fully comprehended despite the implication of various factors such as inflammation, hormones, metabolism and immunology (4). Recently, emerging evidence suggests that metabolic disturbances play a vital role in the development and progression of endometriosis (5, 6). In particular, dysregulation of glucose and lipid metabolism has been observed in women with endometriosis (7–9).

The triglyceride-glucose (TyG) index, derived from the fasting triglyceride and glucose levels, has been considered as a novel and reliable marker for assessing insulin resistance and shows comparable effectiveness to the commonly used homeostatic model assessment (HOMA) insulin resistance index (10). Due to its advantages of economic benefits and easy availability, the TyG index has gained attention as a comprehensive surrogate measure of insulin resistance.

Multiple epidemiological studies have demonstrated the association of TyG with a range of diseases, such as type 2 diabetes (11), cardiovascular disease (12), metabolic dysfunction-associated fatty liver disease (13) and gynecologic cancers (14, 15). Given the potential involvement of metabolic disturbances in endometriosis pathogenesis, it is plausible to explore the association of insulin resistance with the risk of endometriosis. However, to our knowledge, the association between the TyG index and endometriosis has not been explored till date.

This population-based study aims to investigate the association between the TyG index and the risk of endometriosis using the nationally representative American population from the National Health and Nutrition Examination Survey (NHANES) (16), to provide comprehensive epidemiological evidence for the clinical role of the TyG index in the risk assessment and prevention of endometriosis.

## 2 Materials and methods

### 2.1 Data source and population

**Figure 1:**
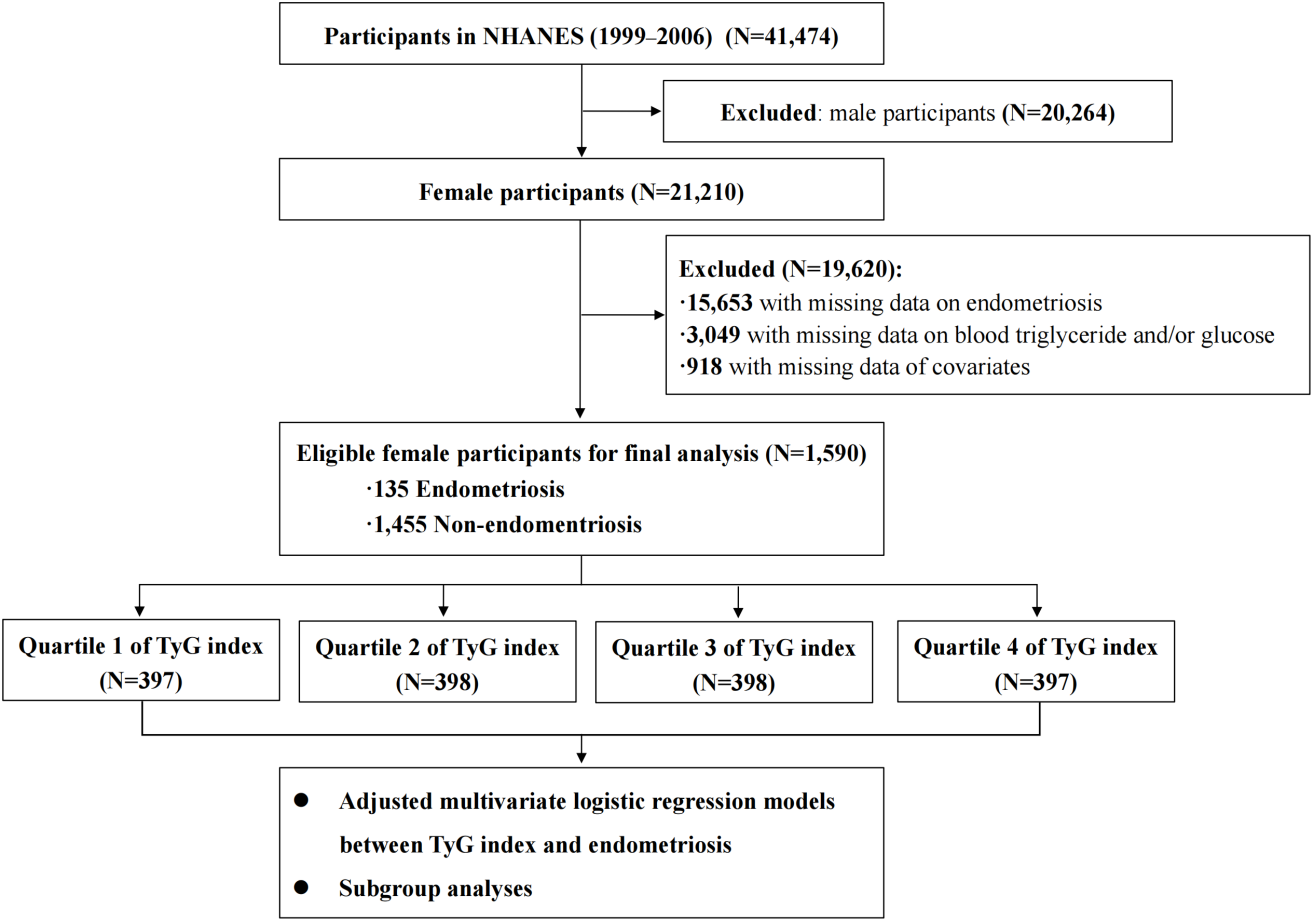
The flowchart of this study. NHANES, National Health and Nutrition Examination Survey; TyG index, triglyceride-glucose index.

The NHANES program is designed to assess the health and nutritional status of the American population through a series of interviews, physical examinations and laboratory tests. It is conducted by the Centers for Disease Control and Prevention (16) in the United States (US). In this current study, we collected publicly available data on a total of 21,210 female participants from NHANES (1999–2006). We excluded 15,653 participants with missing data on the diagnosis of endometriosis, and 3,049 participants with missing laboratory data on fasting blood triglyceride and fasting glucose levels. Additionally, 918 participants who had incomplete data on the potential confounding variables (described below) were also excluded. Consequently, a total of 1,590 female participants were included in the final analysis. The study flowchart is presented in **Figure 1**.

### 2.2 Ascertainment of endometriosis

The diagnosis of endometriosis was based on the “Questionnaire on Reproductive Health” at each survey circle in NHANES between 1999 and 2006. Participants who reported “yes” to the question “Told by the doctor having endometriosis?” were identified to have endometriosis.

### 2.3 TyG index and covariates

The TyG index was calculated using the formula Ln [fasting triglyceride (mg/dL) × fasting glucose (mg/dL)/2]. The data on fasting triglycerides and fasting glucose were obtained from the “Laboratory Data” in NHANES. In this study, the TyG Index ranged from 7.049 to 11.951, representing a continuous value.

Based on the previous studies (17, 18) and clinical experience, the following covariates were included in the analysis: age (years), ethnicity (non-Hispanic White, non-Hispanic Black, Mexican Americans, and other race), educational level (above high school, high school graduate, and less than high school), marital status (never married, married, and other), fertility status (≥ one birth or nulliparous), body mass index (BMI), diabetes (no or yes), drinking status (current drinker, former drinker, and non-drinker), smoking status (current smoker, former smoker, and non-smoker) and the use of oral contraceptives (no or yes).

BMI was determined by dividing the weight in kilograms by the square of height in meters (kg/m^2^). Diabetes was assessed based on the following criteria: being told diabetes by a doctor, glycated hemoglobin A1c exceeding 6.5%, fasting glucose equal to or greater than 7.0mmol/L, random blood glucose equal to or greater than 11.1 mmol/L, blood glucose equal to or greater than11.1mmol/L during a 2-hour oral glucose tolerance test, and the use of diabetes medication or insulin.

### 2.4 Statistical analysis

All analyses were conducted in accordance with the NHANES analytic guidelines. Categorical variables were compared by the χ^2^ test, and continuous variables were compared by the t-test or Wilcoxon rank sum nonparametric test based on the results of the normality test. The participants were categorized into four groups (Q1, Q2, Q3, Q4) according to the quartiles of the TyG index, using the Q1 group as the reference group. We utilized multivariate logistic regression models to calculate the odd ratios (OR) and 95% confidence intervals (CI) to assess the relationship between the TyG index and endometriosis. Initially, a crude model was applied, considering only the TyG index. Subsequently, three adjusted models were adopted. Model 1 was adjusted for age, ethnicity, education level and marital status. In Model 2, we further adjusted for BMI, diabetes, and fertility status in addition to the covariates included in Model 1. Lastly, Model 3 accounted for drinking status, smoking status, and use of oral contraceptives in addition to the covariates included in Model 2. Additionally, we estimated linear trends across quartiles of the TyG index by treating the median value in each quartile as a continuous variable in regression models. Moreover, we examined the association of the TyG index with the risk of endometriosis on a continuous scale using the restricted cubic spline (RCS) curve based on the logistic regression model. Subgroup analyses were performed stratified by fertility status, diabetes, drinking status, smoking status and the usage of oral contraceptives.

All statistical analyses were conducted using R software (version 4.2.2). All *P* values were two-sided with *P*<0.05 considered as statistically significant.

## 3 Results

### 3.1 Characteristics of the participants

The baseline characteristics between endometriosis and non-endometriosis groups are presented in **Table 1**. Among the 1,590 eligible participants, 135 (8.5%) women were diagnosed with endometriosis. The mean age of participants in the entire study was 39.20, ranging from 20 to 54 years. Participants diagnosed with endometriosis were found to be older, had a higher level of education, and were more likely to be a smokers. Furthermore, there were significant differences in ethnicity between the two groups.

**Table 1:** Baseline characteristics between endometriosis and non-endometriosis groups.

Characteristics	Total (n=1,590)	Endometriosis (n=135)	Non-Endometriosis (n=1,455)	*P* value
**Age (years), mean (SD)**	39.20 (9.26)	41.96 (7.85)	38.95 (9.34)	<0.001
**Ethnicity, n (%)**				<0.001
Mexican American	367 (23.1)	9 (6.7)	358 (24.6)	
Non-Hispanic Black	382 (24.0)	25 (18.5)	357 (24.5)	
Non-Hispanic White	702 (44.2)	94 (69.6)	608 (41.8)	
Other Races	139 (8.7)	7 (5.2)	132 (9.1)	
**Education level, n (%)**				0.001
Less than high school	425 (26.7)	19 (14.1)	406 (27.9)	
High school graduate	374 (23.5)	42 (31.1)	332 (22.8)	
Above high school	791 (49.7)	74 (54.8)	717 (49.3)	
**Marital status, n (%)**				0.254
Never married	185 (11.6)	10 (7.4)	175 (12.0)	
Married	955 (60.1)	87 (64.4)	868 (59.7)	
Other	450 (28.3)	38 (28.1)	412 (28.3)	
**Fertility status, n (%)**				0.192
Nulliparous	95 (6.0)	12 (8.9)	83 (5.7)	
≥one birth	1495 (94.0)	123 (91.1)	1372 (94.3)	
**BMI, mean (SD)**	29.18 (7.36)	28.89 (6.66)	29.20 (7.43)	0.635
**Diabetes, n (%)**				0.793
No	1469 (92.4)	126 (93.3)	1343 (92.3)	
Yes	121 (7.6)	9 (6.7)	112 (7.7)	
**Smoking status, n (%)**				<0.001
Never	926 (58.2)	57 (42.2)	869 (59.7)	
Former	260 (16.4)	30 (22.2)	230 (15.8)	
Now	404 (25.4)	48 (35.6)	356 (24.5)	
**Drinking status, n (%)**				0.127
Never	255 (16.0)	15 (11.1)	240 (16.5)	
Former	278 (17.5)	30 (22.2)	248 (17.0)	
Now	1057 (66.5)	90 (66.7)	967 (66.5)	
**Oral contraceptive, n (%)**				0.054
No	319 (20.1)	18 (13.3)	301 (20.7)	
Yes	1271 (79.9)	117 (86.7)	1154 (79.3)	
**Fasting glucose (mg/dl), median [IQR]**	93.0 [87.0, 100.0]	92.3 [86.9, 100.4]	93.0 [87.1, 99.8]	0.935
**Fasting triglyceride (mg/dl), median [IQR]**	100.0 [70.0, 146.0]	119.0 [77.0, 179.0]	99.0 [69.0, 143.0]	0.001
**TyG index, mean (SD)**	8.51 (0.62)	8.67 (0.64)	8.49 (0.62)	0.002
**TyG index Quartile, n (%)**				0.029
Q1	397 (25.0)	24 (17.8)	373 (25.6)	
Q2	398 (25.0)	31 (23.0)	367 (25.2)	
Q3	398 (25.0)	33 (24.4)	365 (25.1)	
Q4	397 (25.0)	47 (34.8)	350 (24.1)	

IQR, interquartile range; SD, standard deviation; TyG index, triglyceride-glucose index; BMI, body mass index.

In the overall study population, the mean TyG index was 8.51, with a significantly higher mean TyG index in cases with endometriosis compared to those without endometriosis (8.67 versus 8.49, *P*=0.002). Furthermore, a higher percentage of the highest quartile (Q4) TyG index (34.8% versus 24.1%) and a lower percentage of the lower quartile (Q1) TyG index (17.8% versus 25.6%) were found in the endometriosis group than the non-endometriosis group. Meanwhile, the baseline characteristics of the participants based on quartile categories of the TyG index are shown in **Supplementary Table S1**.

### 3.2 Association of the TyG index with endometriosis

The associations of TyG index with endometriosis in the overall cohort, are presented in **Table 2**. In the unadjusted model, the OR for participants with endometriosis in the highest quartile of the TyG index was 2.09 (95% CI: 1.25–3.49) compared to those in the lowest quartile (*P* for trend = 0.004). Similarly, in adjusted multivariate models 1, 2 and 3, the positive association of the TyG index with the risk of endometriosis persisted. After fully adjusting for the potential confounders, participants in the highest quartile of TyG had a 104% higher risk of endometriosis compared to those in the lowest quartile (OR _Q4 versus Q1_ 2.04, 95% CI: 1.15–3.62). Additionally, significant increasing trends in the adjusted odds of endometriosis across the quartiles of the TyG index were observed in all models (Model 1: *P* for trend=0.010, Model 2: *P* for trend=0.004, Model 3: *P* for trend=0.013).

**Table 2:** Associations between the TyG index and endometriosis in the total cohort.

TyG index	Cases with endometriosis/N	OR (95% CI)			
		Crude ^a^	Model 1 ^b^	Model 2 ^c^	Model 3 ^d^
**Continuous Values** (7.049–11.951)	135/1,590	1.52 (1.17–1.97)^*^	1.53 (1.15–2.03)^*^	1.73 (1.26–2.36)^*^	1.62 (1.18–2.22)^*^
Quartile Categories					
Q1 (7.049–8.061)	24/397	Reference	Reference	Reference	Reference
Q2 (8.063–8.452)	31/398	1.31 (0.76–2.28)	1.29 (0.74–2.25)	1.35 (0.76–2.37)	1.26 (0.71–2.23)
Q3 (8.453–8.865)	33/398	1.41 (0.81–2.42)	1.35 (0.77–2.34)	1.42 (0.81–2.51)	1.31 (0.74–2.32)
Q4 (8.866–11.951)	47/397	2.09 (1.25–3.49)^*^	1.99 (1.16–3.40)^*^	2.27 (1.29–4.00)^*^	2.04 (1.15–3.62)^*^
*P* for trend		0.004	0.010	0.004	0.013

*Statistically significant association.

a Crude model was adjusted for nothing.

b Model 1 was adjusted for age, ethnicity, education level and marital status.

c Model 2 included the covariates of Model 1 with additional adjustment for diabetes, body mass index and fertility status.

d Model 3 included the covariates of Model 2 with additional adjustment for drinking status, smoking status and use of oral contraceptives.

TyG index, triglyceride-glucose index; OR, odds ratio; CI, confidence interval.

**Figure 2:**
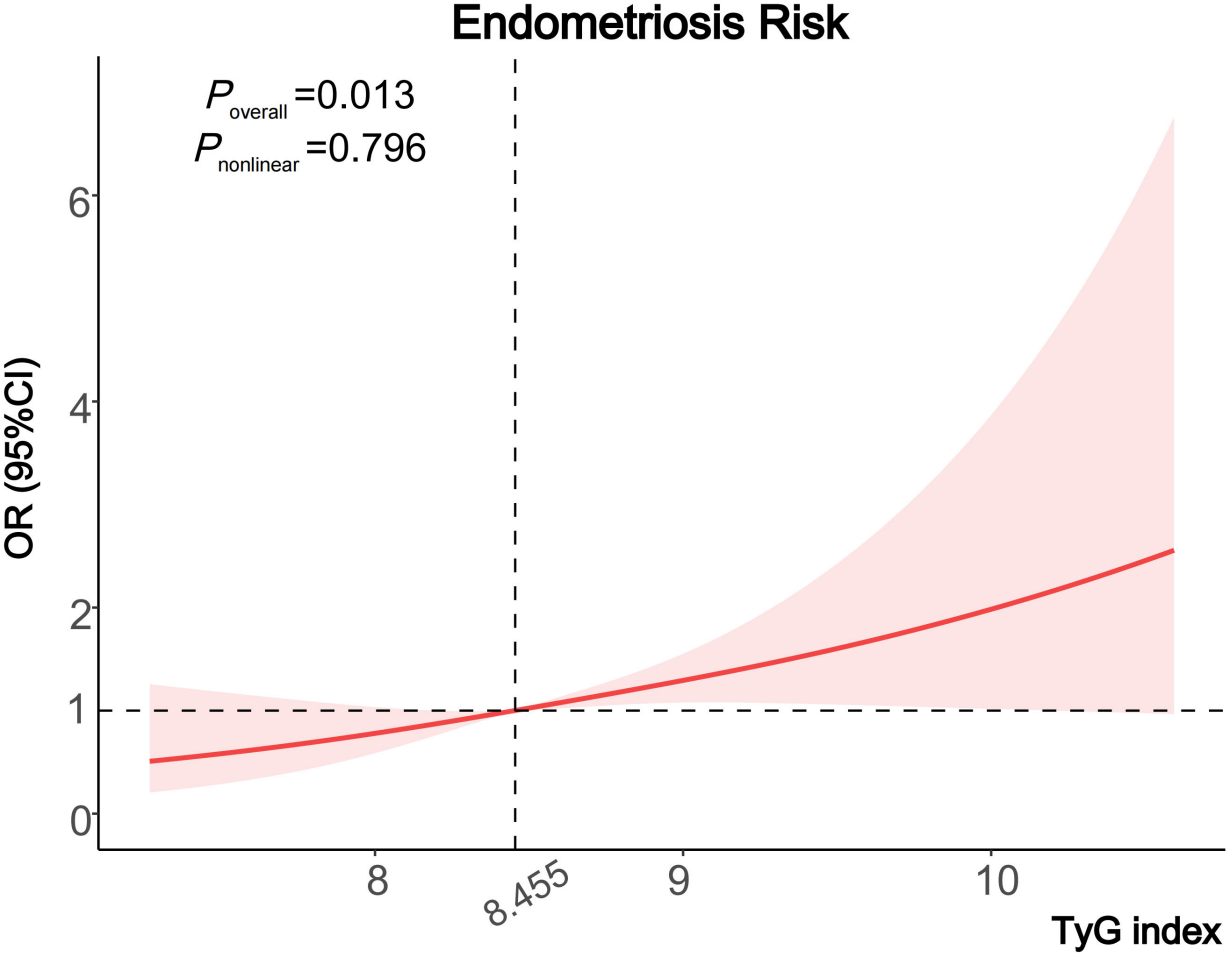
Cubic regression spline of the endometriosis risk by TyG index among the entire population. Cubic regression spline was adjusted for age, ethnicity, education level, marital status, fertility status, body mass index, diabetes, drinking status, smoking status and the use of oral contraceptives. ORs are indicated by solid lines and 95% CIs are presented by shaded areas. TyG index, triglyceride-glucose index; OR, odds ratio; CI, confidence interval.

When assessing the association between the TyG index and the risk of endometriosis on a continuous scale, the fully adjusted logistic regression model also conveyed that the TyG index was positively related to the endometriosis (OR 1.62, 95% CI: 1.18–2.22) (**Table 2**). Moreover, we also observed a dose-response correlation between the TyG index and the endometriosis risk by the RCS analysis (*P*=0.013) (**Figure 2**).

### 3.3 Subgroup analysis

**Figure 3:**
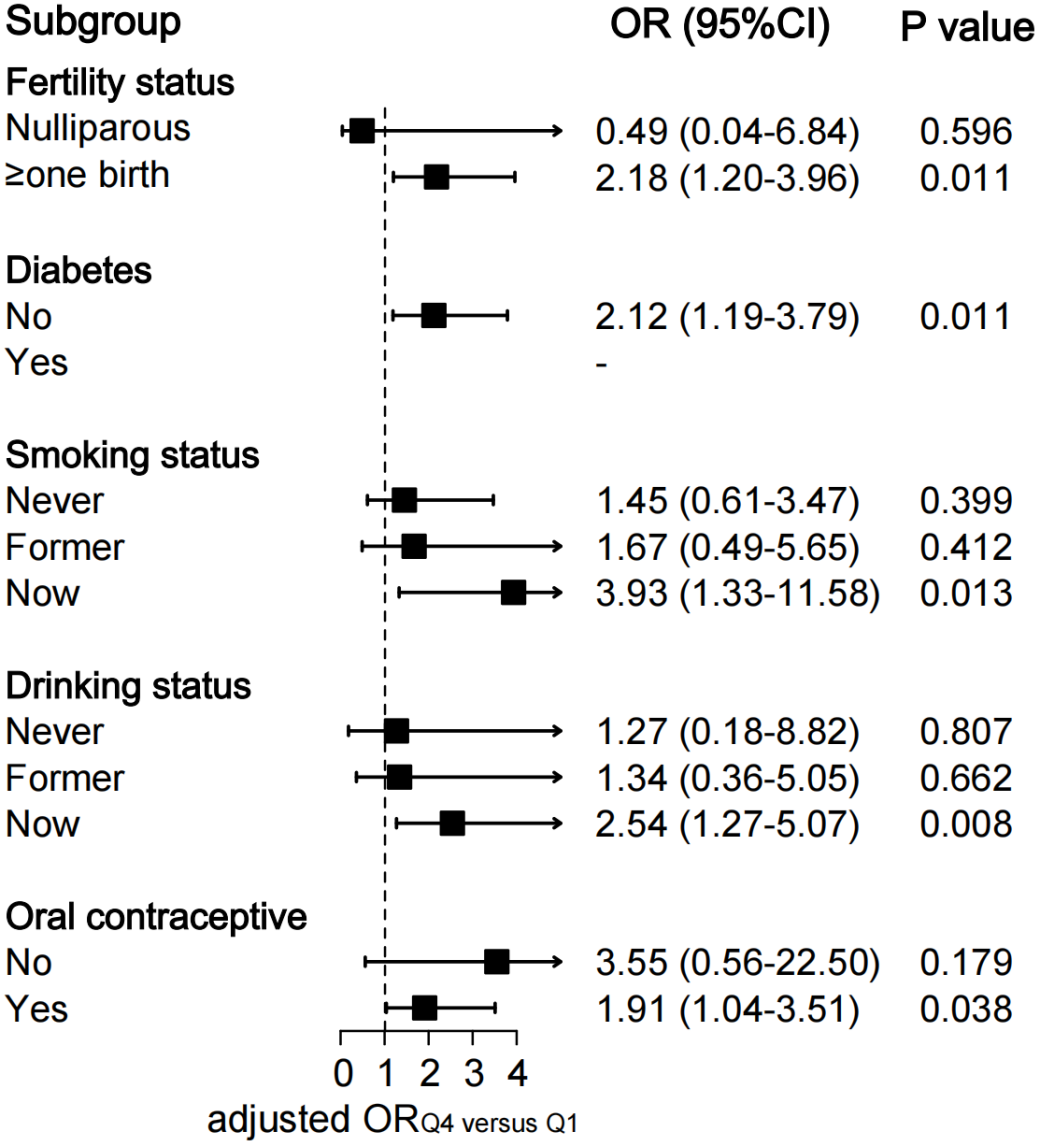
Subgroup analysis of association between quartiles of TyG index and endometriosis. The ORs were assessed by multivariate logistic regression models adjusted for age, ethnicity, education level, marital status, fertility status, body mass index, diabetes, drinking status, smoking status and the use of oral contraceptives. All Models were not adjusted for the stratified covariates on which the subgroup analyses were conducted. TyG index, triglyceride-glucose index; OR, odds ratio; CI, confidence interval.

To explore the association of the TyG index with the risk of endometriosis in different populations, we performed in-depth subgroup analyses stratified by fertility status, diabetes, smoking status, drinking status, and usage of oral contraceptives (**Figure 3**; **Supplementary Table S2**).

The fully-adjusted logistic regression models also revealed a significant positive relationship between quartiles of TyG index and the risk of endometriosis in parous participants (OR _Q4 versus Q1_ 2.18, 95% CI: 1.20–3.96; *P*=0.011), in participants without diabetes (OR _Q4 versus Q1_ 2.12, 95% CI: 1.19–3.79; *P*=0.011), in participants who smoke currently (OR _Q4 versus Q1_ 3.93, 95% CI: 1.33–11.58; *P*=0.013), in participants who drink currently (OR _Q4 versus Q1_ 2.54, 95% CI: 1.27–5.07; *P*=0.008), and in participants who use oral contraceptives (OR _Q4 versus Q1_ 1.91, 95% CI: 1.04–3.51; *P*=0.038) (**Figure 3**). Significant increasing trends in the odds of endometriosis across the quartiles of the TyG index were observed in the above-mentioned subgroups (all *P* for trend <0.05) (**Supplementary Table S2**).

## 4 Discussion

To the best of our knowledge, this nationally representative study represents the first investigation into the correlation between the TyG index and the risk of endometriosis. We found a significantly positive association of the TyG index with the risk of endometriosis in the US adult population, highlighting the clinical role of the TyG index on the prevention and management strategies for endometriosis.

As a multifactorial and systemic disease, endometriosis is caused by many factors and the exact pathogenesis has not been clearly explained (5). Recently, metabolic abnormalities, including dyslipidemia and glucose metabolism dysfunction, were reported to be an increasingly significant etiology of endometriosis, with the development of metabolomics technology (6–9).

TyG index, measured based on blood fasting triglycerides and glucose, has been recommended as an effective surrogate marker for insulin resistance, and could integrate the effect of both triglycerides as well as glucose on endometriosis (19). Numerous studies have demonstrated the potential of the TyG index in predicting the risk of various metabolism-related disorders (11–15). However, the relationship between the TyG index and endometriosis has not been explored yet.

In our study, we observed a 104% higher risk of endometriosis in participants within the highest quartile of the TyG index, compared to those in the lowest quartile, after adjusting for various covariates. These findings provided novel insights into the association between insulin resistance and the development of endometriosis, suggesting that the TyG index could potentially serve as a valuable clinical tool for predicting and assessing the risk of endometriosis.

The potential underlying mechanisms linking the TyG index to endometriosis are still not well understood and there might be several possible explanations as follows. First, insulin resistance could lead to hyperinsulinemia and elevated levels of insulin-like growth factor-1 (IGF-1), which may potentially promote the growth and proliferation of endometrial tissue outside the uterus (20, 21). Additionally, insulin resistance is associated with chronic systematic inflammation, which has been implicated in the development and progression of endometriosis (1, 22). Moreover, hyperinsulinemia might promote the production of androgens, leading to hormonal imbalances between estrogen and progesterone, which may further facilitate the establishment and growth of ectopic endometrial lesions (23, 24).

In the stratified analysis of our study, we observed variations in the association between the TyG index and the risk of endometriosis across different subgroups. When stratified by fertility status, a significant relationship of TyG index with endometriosis was observed in participants who had given birth but not in nulliparous women. It is well-established that pregnancy could induce significant metabolic and hormonal changes, which might modulate the impact of insulin resistance on the progression of endometriosis (25). Meanwhile, parity has been regarded as an important protective factor for endometriosis (26). It is possible that the effect of insulin resistance on endometriosis might be attenuated among nulliparous women in our study.

Notably, in the subgroup analysis stratified by the smoking and drinking status, we found a considerable 3.93 and 2.54 times risk of endometriosis in women within the highest quartiles of the TyG index than those within the lowest quartiles, among women who smoke or drink currently, respectively. This observed phenomenon could potentially be ascribed to the hypothesis that smoking and alcohol consumption could exacerbate the adverse impact of insulin resistance on endometrial tissue, ultimately resulting in an increased susceptibility to the development of endometriosis (27, 28). Our findings conveyed that monitoring the TyG index could potentially be a useful tool in identifying individuals at a higher risk for endometriosis among the American female population who engage in smoking and alcohol consumption.

Regarding the stratified analysis by the use of oral contraceptives, a previous meta-analysis reported use of oral contraceptives could reduce the risk of endometriosis, which demonstrated that the use of oral contraceptives might serve as a protective factor against the development of endometriosis (29). In the present study, we also found a significantly positive correlation between the TyG index and the risk of endometriosis among those who used oral contraceptives. Nevertheless, no significant relationship was found in the women without the use of oral contraceptives. Prospective researches are necessary to further investigate the exact relationship between the TyG index and endometriosis in these specific women groups.

### 4.1 Strengths and limitations

The strengths of this study include its population-based dataset from NHANES, allowing the generalizability of our findings in the US population. Meanwhile, to explore the association of the TyG index with endometriosis preciously and thoroughly, in-depth adjustments for potential confounders and subgroups were performed, which could lead to more robust results. Moreover, to our knowledge, this study was the first to provide insights into the association of insulin resistance with the risk of endometriosis, highlighting the significant value of the TyG index in the risk assessment of endometriosis. We recommended that the TyG index should be calculated and listed routinely in laboratory biochemical tests, which could help gynecologists effectively identify individuals at a higher risk and facilitate early intervention and treatment. Additionally, targeting metabolic dysfunction, such as insulin resistance, through lifestyle modifications and pharmacological interventions, may present a novel therapeutic approach to managing endometriosis.

However, several limitations should be considered. First, the cross-sectional nature of the study limits the establishment of a causal relationship between the TyG index and endometriosis. Second, we relied on self-reported diagnosis of endometriosis in the NHANES, which might introduce recall bias. Prospective longitudinal studies are warranted to further confirm these findings.

## 5 Conclusion

To conclude, this nationally representative study found that elevated insulin resistance, as reflected by a higher TyG index, was associated with a higher risk of endometriosis in American adults. Our findings suggested TyG index may serve as an alternative tool to predict the risk of endometriosis, and potentially guide future prevention strategies. Further studies are needed to confirm these findings and elucidate the underlying mechanisms.

## Data availability statement

The datasets presented in this study can be found in online repositories. The names of the repository/repositories and accession number(s) can be found below: National Health and Nutrition Examination Survey (https://www.cdc.gov/nchs/nhanes).

## Ethics statement

The studies involving humans were approved by National Center for Health Statistics Ethics Review Board. The studies were conducted in accordance with the local legislation and institutional requirements. The participants provided their written informed consent to participate in this study.

## Author contributions

PL: Data curation, Formal analysis, Methodology, Software, Writing – original draft, Writing – review & editing. YW: Investigation, Writing – review & editing. XJ: Investigation, Writing – review & editing. WK: Formal analysis, Software, Writing – review & editing. ZP: Visualization, Writing – review & editing. CX: Validation, Writing – review & editing. YG: Validation, Writing – review & editing. JM: Conceptualization, Data curation, Funding acquisition, Supervision, Writing – review & editing.

## Supplementary material

Supplementary Table 1 Baseline characteristics according to quartile categories of TyG index.

Supplementary Table 2 Subgroup analysis of the association between quartiles of TyG index and endometriosis.

## References

[1] Taylor HS Kotlyar AM Flores VA. Endometriosis is a chronic systemic disease: clinical challenges and novel innovations. Lancet. (2021) 397:839–52. doi: 10.1016/S0140-6736(21)00389-5 33640070

[2] Giudice LC Horne AW Missmer SA. Time for global health policy and research leaders to prioritize endometriosis. Nat Commun. (2023) 14:8028. doi: 10.1038/s41467-023-43913-9 38049392 PMC10696045

[3] Simoens S Dunselman G Dirksen C Hummelshoj L Bokor A Brandes I. The burden of endometriosis: costs and quality of life of women with endometriosis and treated in referral centres. Hum Reprod. (2012) 27:1292–9. doi: 10.1093/humrep/des073 22422778

[4] Zondervan KT Becker CM Missmer SA. Endometriosis. N Engl J Med. (2020) 382:1244–56. doi: 10.1056/NEJMra1810764 32212520

[5] Horne AW Missmer SA. Pathophysiology, diagnosis, and management of endometriosis. BMJ. (2022) 379:e070750. doi: 10.1136/bmj-2022-070750 36375827

[6] Lu J Ling X Liu L Jiang A Ren C Lu C. Emerging hallmarks of endometriosis metabolism: A promising target for the treatment of endometriosis. Biochim Biophys Acta Mol Cell Res. (2023) 1870:119381. doi: 10.1016/j.bbamcr.2022.119381 36265657

[7] Li B Zhang Y Zhang L Zhang L. Association between endometriosis and metabolic syndrome: a cross-sectional study based on the National Health and Nutrition Examination Survey data. Gynecol Endocrinol. (2023) 39:2254844. doi: 10.1080/09513590.2023.2254844 37673102

[8] Lu C Qiao P Fu R Wang Y Lu J Ling X. Phosphorylation of PFKFB4 by PIM2 promotes anaerobic glycolysis and cell proliferation in endometriosis. Cell Death Dis. (2022) 13:790. doi: 10.1038/s41419-022-05241-6 36109523 PMC9477845

[9] Melo AS Rosa-e-Silva JC Rosa-e-Silva A Poli-Neto OB Ferriani RA Vieira CS. Unfavorable lipid profile in women with endometriosis. Fertil Steril. (2010) 93:2433–6. doi: 10.1016/j.fertnstert.2009.08.043 19969295

[10] Guerrero-Romero F Simental-Mendía LE González-Ortiz M Martínez-Abundis E Ramos-Zavala MG Hernández-González SO. The product of triglycerides and glucose, a simple measure of insulin sensitivity. Comparison with the euglycemic-hyperinsulinemic clamp. J Clin Endocrinol Metab. (2010) 95:3347–51. doi: 10.1210/jc.2010-0288 20484475

[11] Park B Lee HS Lee Y-J. Triglyceride glucose (TyG) index as a predictor of incident type 2 diabetes among nonobese adults: a 12-year longitudinal study of the Korean Genome and Epidemiology Study cohort. Transl Res. (2021) 228:42–51. doi: 10.1016/j.trsl.2020.08.003 32827706

[12] Jung M-H Yi S-W An SJ Yi J-J Ihm S-H Han S. Associations between the triglyceride-glucose index and cardiovascular disease in over 150,000 cancer survivors: a population-based cohort study. Cardiovasc Diabetol. (2022) 21:52. doi: 10.1186/s12933-022-01490-z 35429972 PMC9013459

[13] Wang J Yan S Cui Y Chen F Piao M Cui W. The diagnostic and prognostic value of the triglyceride-glucose index in metabolic dysfunction-associated fatty liver disease (MAFLD): A systematic review and meta-analysis. Nutrients. (2022) 14:4969. doi: 10.3390/nu14234969 36500999 PMC9741077

[14] Shi H Zhou L Yang S Zhou H. The relationship between Triglyceride and glycose (TyG) index and the risk of gynaecologic and breast cancers. Clin Nutr ESPEN. (2022) 51:345–52. doi: 10.1016/j.clnesp.2022.08.004 36184226

[15] Shi H Guo F Zheng K Li R Zhou H. Triglyceride-glucose index (TyG index) and endometrial carcinoma risk: A retrospective cohort study. Int J Gynaecol Obstet. (2024) 164:298–304. doi: 10.1002/ijgo.15038 37555382

[16] Centers for Disease Control and Prevention. About the National Health and Nutrition Examination Survey . Available online at: http://www.cdc.gov/nchs/nhanes/about_nhanes.htm.

[17] Vercellini P Viganò P Somigliana E Fedele L. Endometriosis: pathogenesis and treatment. Nat Rev Endocrinol. (2014) 10:261–75. doi: 10.1038/nrendo.2013.255 24366116

[18] Shafrir AL Farland LV Shah DK Harris HR Kvaskoff M Zondervan K. Risk for and consequences of endometriosis: A critical epidemiologic review. Best Pract Res Clin Obstet Gynaecol. (2018) 51:1–15. doi: 10.1016/j.bpobgyn.2018.06.001 30017581

[19] Brito A Hermsdorff HHM Filgueiras MDS Suhett LG Vieira-Ribeiro SA Franceschini S. Predictive capacity of triglyceride-glucose (TyG) index for insulin resistance and cardiometabolic risk in children and adolescents: a systematic review. Crit Rev Food Sci Nutr. (2021) 61:2783–92. doi: 10.1080/10408398.2020.1788501 32744083

[20] Zhou Y Zeng C Li X Wu P-L Yin L Yu X-L. IGF-I stimulates ERβ and aromatase expression via IGF1R/PI3K/AKT-mediated transcriptional activation in endometriosis. J Mol Med (Berl). (2016) 94:887–97. doi: 10.1007/s00109-016-1396-1 26899323

[21] Kim JG Suh CS Kim SH Choi YM Moon SY Lee JY. Insulin-like growth factors (IGFs), IGF-binding proteins (IGFBPs), and IGFBP-3 protease activity in the peritoneal fluid of patients with and without endometriosis. Fertil Steril. (2000) 73:996–1000. doi: 10.1016/s0015-0282(00)00493-3 10785227

[22] Rohm TV Meier DT Olefsky JM Donath MY. Inflammation in obesity, diabetes, and related disorders. Immunity. (2022) 55:31–55. doi: 10.1016/j.immuni.2021.12.013 35021057 PMC8773457

[23] Lin Y-K Li Y-Y Li Y Li D-J Wang X-L Wang L. SCM-198 prevents endometriosis by reversing low autophagy of endometrial stromal cell via balancing ERα and PR signals. Front Endocrinol. (2022) 13:858176. doi: 10.3389/fendo.2022.858176 PMC9245568 35784569

[24] Maturana MA Spritzer PM. Association between hyperinsulinemia and endogenous androgen levels in peri- and postmenopausal women. Metabolism. (2002) 51:238–43. doi: 10.1053/meta.2002.29997 11833055

[25] De Paoli M Zakharia A Werstuck GH. The role of estrogen in insulin resistance: A review of clinical and preclinical data. Am J Pathol. (2021) 191:1490–8. doi: 10.1016/j.ajpath.2021.05.011 34102108

[26] Peterson CM Johnstone EB Hammoud AO Stanford JB Varner MW Kennedy A. Risk factors associated with endometriosis: importance of study population for characterizing disease in the ENDO Study. Am J Obstet Gynecol. (2013) 208:451.e1–11. doi: 10.1016/j.ajog.2013.02.040 PMC4114145 23454253

[27] Jeong SH Joo HJ Kwon J Park E-C. Association between smoking behavior and insulin resistance using triglyceride-glucose index among South Korean adults. J Clin Endocrinol Metab. (2021) 106:e4531–41. doi: 10.1210/clinem/dgab399 34160623

[28] Tatsumi Y Morimoto A Asayama K Sonoda N Miyamatsu N Ohno Y. Association between alcohol consumption and incidence of impaired insulin secretion and insulin resistance in Japanese: The Saku study. Diabetes Res Clin Pract. (2018) 135:11–7. doi: 10.1016/j.diabres.2017.10.021 29111281

[29] Vercellini P Eskenazi B Consonni D Somigliana E Parazzini F Abbiati A. Oral contraceptives and risk of endometriosis: a systematic review and meta-analysis. Hum Reprod Update. (2011) 17:159–70. doi: 10.1093/humupd/dmq042 20833638

