# Supplementary material for: Association between triglyceride-glucose index and risk of endometriosis in US population: results from the national health and nutrition examination survey (1999–2006)
Source: Front Endocrinol (Lausanne). 2024 May 28;15:1371393. doi: 10.3389/fendo.2024.1371393 (PMC11165080; doi:10.3389/fendo.2024.1371393)
Supplement: Supplementary Table 1 — Baseline characteristics according to quartile categories of TyG index. [file Table_1.docx]

Supplementary Material

**Table S1 Baseline characteristics according to quartile categories of TyG index.**

| **Characteristics** | **Total**  **(n=1,590)** | **TyG Index** | | | | ***P* value** |
| --- | --- | --- | --- | --- | --- | --- |
|  |  | **Q1** **(n=397)** | **Q2 (n=398)** | **Q3 (n=398)** | **Q4 (n=397)** |  |
| **Age (years), mean (SD)** | 39.20 (9.26) | 36.74 (9.21) | 38.44 (8.97) | 39.24 (9.31) | 42.40 (8.65) | <0.001 |
| **Ethnicity, n (%)** |  |  |  |  |  | <0.001 |
| Mexican American | 367 (23.1) | 62 (15.6) | 77 (19.3) | 107 (26.9) | 121 (30.5) |  |
| Non-Hispanic Black | 382 (24.0) | 139 (35.0) | 112 (28.1) | 71 (17.8) | 60 (15.1) |  |
| Non-Hispanic White | 702 (44.2) | 168 (42.3) | 170 (42.7) | 187 (47.0) | 177 (44.6) |  |
| Other Races | 139 (8.7) | 28 (7.1) | 39 (9.8) | 33 (8.3) | 39 (9.8) |  |
| **Education level, n (%)** |  |  |  |  |  | <0.001 |
| Less than high school | 425 (26.7) | 76 (19.1) | 98 (24.6) | 102 (25.6) | 149 (37.5) |  |
| High school graduate | 374 (23.5) | 84 (21.2) | 102 (25.6) | 98 (24.6) | 90 (22.7) |  |
| Above high school | 791 (49.7) | 237 (59.7) | 198 (49.7) | 198 (49.7) | 158 (39.8) |  |
| **Marital status, n (%)** |  |  |  |  |  | <0.001 |
| Never married | 185 (11.6) | 66 (16.6) | 54 (13.6) | 40 (10.1) | 25 (6.3) |  |
| Married | 955 (60.1) | 224 (56.4) | 232 (58.3) | 252 (63.3) | 247 (62.2) |  |
| Other | 450 (28.3) | 107 (27.0) | 112 (28.1) | 106 (26.6) | 125 (31.5) |  |
| **Fertility status, n (%)** |  |  |  |  |  | 0.153 |
| Nulliparous | 95 (6.0) | 33 (8.3) | 20 (5.0) | 20 (5.0) | 22 (5.5) |  |
| ≥one birth | 1495 (94.0) | 364 (91.7) | 378 (95.0) | 378 (95.0) | 375 (94.5) |  |
| **BMI, mean (SD)** | 29.18 (7.36) | 25.79 (6.24) | 29.10 (7.41) | 29.78 (7.28) | 32.03 (7.11) | <0.001 |
| **Diabetes, n (%)** |  |  |  |  |  | <0.001 |
| No | 1469 (92.4) | 390 (98.2) | 389 (97.7) | 381 (95.7) | 309 (77.8) |  |
| Yes | 121 (7.6) | 7 (1.8) | 9 (2.3) | 17 (4.3) | 88 (22.2) |  |
| **Smoking status, n (%)** |  |  |  |  |  | <0.001 |
| Never | 926 (58.2) | 264 (66.5) | 229 (57.5) | 232 (58.3) | 201 (50.6) |  |
| Former | 260 (16.4) | 62 (15.6) | 70 (17.6) | 63 (15.8) | 65 (16.4) |  |
| Now | 404 (25.4) | 71 (17.9) | 99 (24.9) | 103 (25.9) | 131 (33.0) |  |
| **Drinking status, n (%)** |  |  |  |  |  | 0.005 |
| Never | 255 (16.0) | 55 (13.9) | 62 (15.6) | 63 (15.8) | 75 (18.9) |  |
| Former | 278 (17.5) | 51 (12.8) | 67 (16.8) | 74 (18.6) | 86 (21.7) |  |
| Now | 1057 (66.5) | 291 (73.3) | 269 (67.6) | 261 (65.6) | 236 (59.4) |  |
| **Oral contraceptive, n (%)** |  |  |  |  |  | 0.576 |
| No | 319 (20.1) | 79 (19.9) | 75 (18.8) | 76 (19.1) | 89 (22.4) |  |
| Yes | 1271 (79.9) | 318 (80.1) | 323 (81.2) | 322 (80.9) | 308 (77.6) |  |
| **Fasting glucose (mg/dl), median [IQR]** | 93.0  [87.0, 100.0] | 87.9  [83.6, 93.3] | 92.2  [86.8, 97.2] | 93.0  [88.0, 99.0] | 100.6  [93.0, 111.9] | <0.001 |
| **Fasting triglyceride** **(mg/dl), median [IQR]** | 100.0  [70.0, 146.0] | 57.0  [49.0, 64.0] | 83.0  [76.0, 93.0] | 121.0  [109.0, 136.0] | 194.0  [160.0, 248.0] | <0.001 |
| **TyG index, mean (SD)** | 8.51 (0.62) | 7.79 (0.21) | 8.26 (0.11) | 8.65 (0.12) | 9.34 (0.46) | <0.001 |
| **Endometriosis, n (%)** |  |  |  |  |  | 0.029 |
| Yes | 1455 (91.5) | 373 (94.0) | 367 (92.2) | 365 (91.7) | 350 (88.2) |  |
| No | 135 (8.5) | 24 (6.0) | 31 (7.8) | 33 (8.3) | 47 (11.8) |  |

***Note****:* IQR, interquartile range; SD, standard deviation; TyG index, triglyceride-glucose index.

**Table S2 Subgroup analysis of association between quartiles of TyG index and endometriosis.**

| **Subgroup** | **Cases with endometriosis/N** | **TyG index, OR (95% CI)** | | | |  |
| --- | --- | --- | --- | --- | --- | --- |
|  |  | **Q1**  **(7.049–8.061)** | **Q2**  **(8.063–8.452)** | **Q3**  **(8.453–8.865)** | **Q4**  **(8.866–11.951)** | ***P* for trend** |
| **Fertility status** |  |  |  |  |  |  |
| **Nulliparous** | 12/95 |  |  |  |  |  |
| Crude ^a^ |  | Reference | 4.29 (0.93–19.68) | 1.11 (0.17–7.30) | 0.48 (0.05–4.90) | 0.425 |
| Model 1 ^b^ |  | Reference | 5.06 (0.97–26.33) | 1.33 (0.19–9.13) | 0.58 (0.05–6.70) | 0.612 |
| Model 2 ^c^ |  | Reference | 4.64 (0.86–24.95) | 1.23 (0.18–8.52) | 0.58 (0.04–7.77) | 0.581 |
| Model 3 ^d^ |  | Reference | 4.94 (0.87–28.14) | 1.31 (0.18–9.65) | 0.49 (0.04–6.84) | 0.513 |
| **≥one birth** | 123/1495 |  |  |  |  |  |
| Crude ^a^ |  | Reference | 1.16 (0.64–2.11) | 1.46 (0.82–2.59) | 2.28 (1.33–3.91)^*^ | 0.001 |
| Model 1 ^b^ |  | Reference | 1.12 (0.61–2.06) | 1.38 (0.77–2.47) | 2.14 (1.22–3.74)^*^ | 0.003 |
| Model 2 ^c^ |  | Reference | 1.17 (0.63–2.15) | 1.45 (0.80–2.62) | 2.43 (1.35–4.39)^*^ | 0.001 |
| Model 3 ^d^ |  | Reference | 1.08 (0.59–2.01) | 1.31 (0.72–2.39) | 2.18 (1.20–3.96)^*^ | 0.004 |
|  |  |  |  |  |  |  |
| **Diabetes** |  |  |  |  |  |  |
| **No** | 126/1469 |  |  |  |  |  |
| Crude ^a^ |  | Reference | 1.27 (0.73–2.22) | 1.35 (0.78–2.35) | 2.33 (1.38–3.96)^*^ | 0.001 |
| Model 1 ^b^ |  | Reference | 1.25 (0.71–2.19) | 1.28 (0.73–2.24) | 2.19 (1.27–3.79)^*^ | 0.004 |
| Model 2 ^c^ |  | Reference | 1.32 (0.74–2.33) | 1.36 (0.77–2.41) | 2.36 (1.33–4.20)^*^ | 0.003 |
| Model 3 ^d^ |  | Reference | 1.23 (0.69–2.18) | 1.24 (0.70–2.21) | 2.12 (1.19–3.79)^*^ | 0.010 |
| **Yes** | 9/121 |  |  |  |  |  |
| Crude ^a^ |  | - | - | - | - | - |
| Model 1 ^b^ |  | - | - | - | - | - |
| Model 2 ^c^ |  | - | - | - | - | - |
| Model 3 ^d^ |  | - | - | - | - | - |
|  |  |  |  |  |  |  |
| **Smoking status** |  |  |  |  |  |  |
| **Never** |  |  |  |  |  |  |
| Crude ^a^ | 57/926 | Reference | 1.26 (0.56–2.83) | 1.56 (0.72–3.36) | 1.82 (0.84–3.93) | 0.108 |
| Model 1 ^b^ |  | Reference | 1.28 (0.56–2.91) | 1.49 (0.68–3.29) | 1.66 (0.74–3.73) | 0.203 |
| Model 2 ^c^ |  | Reference | 1.21 (0.52–2.80) | 1.42 (0.63–3.17) | 1.46 (0.61–3.46) | 0.362 |
| Model 3 ^d^ |  | Reference | 1.19 (0.52–2.76) | 1.41 (0.63–3.17) | 1.45 (0.61–3.47) | 0.363 |
| **Former** | 30/260 |  |  |  |  |  |
| Crude ^a^ |  | Reference | 1.38 (0.46–4.12) | 0.98 (0.30–3.23) | 1.50 (0.50–4.49) | 0.581 |
| Model 1 ^b^ |  | Reference | 1.27 (0.42–3.85) | 0.89 (0.27–2.96) | 1.39 (0.45–4.27) | 0.683 |
| Model 2 ^c^ |  | Reference | 1.36 (0.44–4.19) | 0.93 (0.27–3.17) | 1.66 (0.50–5.55) | 0.526 |
| Model 3 ^d^ |  | Reference | 1.33 (0.43–4.15) | 0.83 (0.24–2.90) | 1.67 (0.49–5.65) | 0.547 |
| **Now** | 48/404 |  |  |  |  |  |
| Crude ^a^ |  | Reference | 1.08 (0.37–3.19) | 1.30 (0.46–3.68) | 2.19 (0.84–5.67)^*^ | 0.043 |
| Model 1 ^b^ |  | Reference | 0.97 (0.32–2.90) | 1.19 (0.41–3.48) | 2.46 (0.89–6.83)^*^ | 0.027 |
| Model 2 ^c^ |  | Reference | 1.16 (0.38–3.54) | 1.55 (0.51–4.70) | 3.95 (1.34–11.63)^*^ | 0.003 |
| Model 3 ^d^ |  | Reference | 1.16 (0.38–3.55) | 1.55 (0.51–4.69) | 3.93 (1.33–11.58)^*^ | 0.003 |
|  |  |  |  |  |  |  |
| **Drinking status** |  |  |  |  |  |  |
| **Never** | 15/255 |  |  |  |  |  |
| Crude ^a^ |  | Reference | 1.35 (0.22–8.38) | 1.80 (0.32–10.21) | 2.30 (0.45–11.88) | 0.268 |
| Model 1 ^b^ |  | Reference | 1.4 0(0.20–9.71) | 1.63 (0.27–9.89) | 1.46 (0.24–8.86) | 0.723 |
| Model 2 ^c^ |  | Reference | 1.49 (0.21–10.5) | 1.70 (0.28–10.46) | 1.64 (0.25–10.91) | 0.633 |
| Model 3 ^d^ |  | Reference | 1.25 (0.18–8.80) | 1.39 (0.22–8.92) | 1.27 (0.18–8.82) | 0.831 |
| **Former** | 30/278 |  |  |  |  |  |
| Crude ^a^ |  | Reference | 0.35 (0.08–1.48) | 1.31 (0.45–3.80) | 0.99 (0.34–2.90) | 0.532 |
| Model 1 ^b^ |  | Reference | 0.28 (0.06–1.21) | 1.15 (0.37–3.52) | 0.96 (0.29–3.10) | 0.471 |
| Model 2 ^c^ |  | Reference | 0.32 (0.07–1.47) | 1.52 (0.46–5.03) | 1.28 (0.36–4.61) | 0.285 |
| Model 3 ^d^ |  | Reference | 0.31 (0.07–1.46) | 1.37 (0.40–4.65) | 1.34 (0.36–5.05) | 0.262 |
| **Now** | 90/1057 |  |  |  |  |  |
| Crude ^a^ |  | Reference | 1.76 (0.92–3.38) | 1.27 (0.64–2.55) | 2.60 (1.38–4.88)^*^ | 0.007 |
| Model 1 ^b^ |  | Reference | 1.70 (0.88–3.28) | 1.21 (0.60–2.44) | 2.52 (1.31–4.82)^*^ | 0.012 |
| Model 2 ^c^ |  | Reference | 1.76 (0.91–3.43) | 1.25 (0.61–2.56) | 2.86 (1.44–5.66)^*^ | 0.088 |
| Model 3 ^d^ |  | Reference | 1.64 (0.84–3.21) | 1.18 (0.58–2.43) | 2.54 (1.27–5.07)^*^ | 0.017 |
|  |  |  |  |  |  |  |
| **Oral Contraceptive** |  |  |  |  |  |  |
| **No** | 18/319 |  |  |  |  |  |
| Crude ^a^ |  | Reference | 2.75 (0.52–14.63) | 1.58 (0.26–9.74) | 3.80 (0.78–18.47) | 0.119 |
| Model 1 ^b^ |  | Reference | 2.81 (0.51–15.36) | 1.70 (0.27–10.67) | 4.09 (0.76–22.03) | 0.136 |
| Model 2 ^c^ |  | Reference | 2.87 (0.50–16.37) | 1.65 (0.26–10.6) | 4.19 (0.70–25.14) | 0.178 |
| Model 3 ^d^ |  | Reference | 2.58 (0.44–15.12) | 1.60 (0.24–10.58) | 3.55 (0.56–22.5) | 0.252 |
| **Yes** | 117/1271 |  |  |  |  |  |
| Crude ^a^ |  | Reference | 1.18 (0.65–2.13) | 1.38 (0.78–2.45) | 1.95 (1.13–3.37)^*^ | 0.010 |
| Model 1 ^b^ |  | Reference | 1.16 (0.64–2.10) | 1.26 (0.70–2.26) | 1.78 (1.01–3.16)^*^ | 0.037 |
| Model 2 ^c^ |  | Reference | 1.22 (0.67–2.23) | 1.36 (0.75–2.46) | 1.95 (1.07–3.56)^*^ | 0.023 |
| Model 3 ^d^ |  | Reference | 1.13 (0.62–2.08) | 1.24 (0.68–2.27) | 1.91 (1.04–3.51)^*^ | 0.028 |

***Note****:* ^*^Statistically significant association.

^a^ Crude model was adjusted for nothing.

^b^ Model 1 was adjusted for age, ethnicity, education level and marital status.

^c^ Model 2 included the covariates of Model 1 with additional adjustment for fertility status, body mass index and diabetes.

^d^ Model 3 included the covariates of Model 2 with additional adjustment for drinking status, smoking status and use of oral contraceptives.

All Models were not adjusted for the stratified covariates on which the subgroup analyses were conducted.

TyG index, triglyceride glucose index; OR, odds ratio; CI, confidence interval.
